# The challenge of complexity in evaluating health policies and programs: the case of women’s participatory groups to improve antenatal outcomes

**DOI:** 10.1186/s12913-017-2627-z

**Published:** 2017-09-29

**Authors:** Sara Van Belle, Susan Rifkin, Bruno Marchal

**Affiliations:** 1 Health Policy Unit, Department of Public Health, Institute of Tropical Medicine, Antwerp, Belgium; 2 London School of Hygiene and Tropical Medicine, London, United Kingdom; 3 Health Services Organisation Unit, Department of Public Health, Institute of Tropical Medicine, Antwerp, Belgium

**Keywords:** Impact evaluation, Realist evaluation, Community participation, Empowerment, Complex interventions

## Abstract

**Background:**

During the last years, randomized designs have been promoted as the cornerstone of evidence-based policymaking. Also in the field of community participation, Random Control Trials (RCTs) have been the dominant design, used for instance to examine the contribution of community participation to health improvement. We aim at clarifying why RCTs and related (quasi-) experimental designs may not be the most appropriate approach to evaluate such complex programmes.

**Results:**

We argue that the current methodological debate could be more fruitful if it would start from the position that the choice of designs should fit the nature of the program and research questions rather than be driven by methodological preferences. We present how realist evaluation, a theory-driven approach to research and evaluation, is a relevant methodology that could be used to assess whether and how community participation works. Using the realist evaluation approach to examine the relationship between participation and action of women groups and antenatal outcomes would enable evaluators to examine in detail the underlying mechanisms which influence actual practices and outcomes, as well as the context conditions required to make it work.

**Conclusions:**

Realist research in fact allows opening the black boxes of “community” and “participation” in order to examine the role they play in ensuring cost-effective, sustainable interventions. This approach yields important information for policy makers and programme managers considering how such programs could be implemented in their own setting.

## Background

In the last few years, evaluations of complex interventions have received much attention. In the field of health, the UK Medical Research Council (MRC) produced guidelines for research of complex interventions for the first time in 2000 [1] and updated these in 2008 [2]. These guidelines focused on methods for the assessment of outcomes, of which the random control trial (RCT) is the most dominant. To make evaluations more robust, the MRC recently issued guidelines on how process evaluations can be integrated in evaluations of complex interventions. Their inclusion has the potential to better inform policymakers and practitioners by providing insights into how the intervention was implemented, what mechanisms caused the effects and how context influenced the implementation and results [3].

The current MRC guidance reflects how experimental designs are still considered by many to be the gold standard for evaluation of policies and programs. Indeed, in medicine, they proved very good at demonstrating efficacy of drugs and treatments. Their influence can now also be felt in domains of policymaking, service delivery and programme evaluation. During the last years, randomized designs have been promoted as the cornerstone of evidence-based policymaking, and RCTs have been the dominant design used to examine the contribution of community participation to health improvement [4]. A recent example that has received much attention is the contribution of women’s participatory groups to improved antenatal outcomes. Prost et al. reported on a systematic review and meta-analysis of randomized controlled trials to assess the effect of women groups that practiced variants of participatory learning and action (PLA) [5]. They analysed 7 RCTs carried out in Bangladesh, India, Malawi and Nepal that met their inclusion criteria and concluded that “*with the participation of at least a third of pregnant women and adequate population coverage, women’s groups practising participatory learning and action are a cost-effective strategy to improve maternal and neonatal survival in low-resource settings.*” [5]. The authors acknowledged that it is difficult to attribute the reductions in mortality to specific mechanisms given the complexity of the interventions. However, they combined the results of their meta-analysis with the information from the process evaluations that were carried out within each original study to posit a hypothesis of how participatory action learning leads to the observed effects.

Community participation is a typically messy concept as it can be interpreted in many ways. Standard definitions of “community” and “participation” do not exist [4] and the effect of community participation in terms of improved health outcomes is not easy to assess. Some debate ensued when Rifkin reacted to the paper by Prost et al., expressing her concern that the study reduced the assessment of participatory approaches to an outcome evaluation, ignoring the importance of attitudes, behaviour, power, control, and processes of empowerment, ownership and sustainability [6]. She argued that participation is in essence a process [7], and that the RCT is not the design best suited to evaluate interventions based on PLA.

Prost and colleagues responded that “*the meta-analysis does support a causal relationship between participatory groups and reduced maternal and neonatal deaths. Several mechanisms, acting through both proximal (eg. improved behaviours) and distal (eg, women's empowerment) outcomes, are likely to be implicated*” [8]. They wrote that their meta-analysis contributed to identifying changes in behaviour, which in turn were linked to reduced mortality. The authors ended by inviting “*Rifkin and others who promote participation not to shy away from trials, and to propose new methods for integrating process and impact assessments*”.

We rise to the challenge, aiming at clarifying why RCTs and related experimental designs may not be the most appropriate approach to evaluate complex programs. We will look at the methodological challenges of doing research and evaluation in and of complex problems. We argue that the current methodological debate could be more fruitful if it would start from the position that the choice of designs should be in function of the nature of the program and research questions rather than be driven by methodological preferences. This is in line with the current guidance for health policy and systems research [9]. Evaluation designs following this particular approach have been developed, including realist evaluation (RE). In this paper, we set out to show how RE could be used to assess whether and how community participation works.

## Results

The MRC guidance of 2008 defines an intervention as complex if it contains multiple interacting components, demands some degree of flexibility of implementation, shows a wide range of possible outcomes and a high variability in target population [10]. This definition, however, ignores the key elements of true complex interventions: the role of multiple (synergetic) causal pathways, co-evolution or embeddedness of the intervention in context, path dependence (influence of history and past decisions), emergence (the result of human agency) and co-finality (many potential causes for the outcome). These elements make complex interventions unpredictable (to some degree) and difficult, if not impossible, to reduce to mathematical models. Consequently, establishing the attribution of the outcomes is a major challenge.

Prost et al. report that PLA methods as an intervention to improve antenatal outcomes were used to first ask participants to draw their views of a specific topic and discuss their opinions with each other helped by a facilitator. Focusing on changing attitudes and behaviour of the women this process resulted in agreement by the group of women to prioritize actions to improve antenatal outcomes. Here is a good example of how a particular activity, such facilitated women’s groups, is likely to be implemented in various ways. Group facilitation, for instance, may vary across groups in terms of frequency and place, but also in terms of process, communication techniques, or cultural sensitivity. The effect of group facilitation is likely to be a function of the individual participants’ characteristics, their personal life history and their relations within the family and community, but also of the relations within the group. At the heart of PLA is thus the social interaction between women participating in the program within their structural and cultural context. Consequently, the research design used to understand how the intervention achieves its effects needs to address how emergent processes result from the facilitation. It needs to be able to pick up important context elements, such as other programs aiming at reducing maternal and neonatal mortality, and cultural, economic or political events and changes that may have an influence on empowerment of women (co-evolution).

Researchers using (quasi-)experimental designs typically carry out process evaluations to deal with these issues. The new MRC guidance, for instance, proposes that the process evaluation first establishes the intervention fidelity and “dose”, and then assesses how the intervention is actually carried out across the intervention’s implementation sites, including its reach or coverage [3]. In a second step, the mechanisms of impact are to be established in terms of the participants’ responses to the intervention, mediators and unexpected pathways and outcomes. The guidance suggests that logic models can be used here. Finally, the influence of context on implementation, intervention mechanisms and outcomes is explored. In their paper, Prost et al. similarly draw upon process evaluations to draft a hypothesis in the form of a model (see additional file on the Lancet’s website - http://www.thelancet.com/cms/attachment/2014438397/2035776831/mmc1.pdf).

We agree that process evaluations may provide useful information and can contribute to learning. However, we believe they are not the panacea for the attribution problem in evaluations of complex interventions. Just the terms used in such process evaluations – for instance, ‘fidelity’, ‘mediators’, ‘barriers’ – refer to an approach that seeks to standardize and simplify what are in essence complex dynamic interventions. More importantly, it is not clear, neither from Prost et al.’s paper [5] nor from the MRC guidelines [3], how exactly the process evaluation interfaces with the RCT and how it informs the analysis of data.

Over the last 20 years, there are countercurrents in social sciences to the covering law approach in science. The latter is at the core of experimental designs and in essence seeks to establish the constant conjunction of effect and cause through statistical analysis of associations [11]. In contrast, the mechanism-oriented school focuses on causal mechanisms as the prime component of scientific explanations [12]. It emerged in response to the observation that the covering law approach, unlike in the natural sciences, was far less successful in social sciences where few, if any generally accepted laws were formulated [13].

Mechanism-oriented approaches to causal explanation focus on identifying the mechanism that underlies the intervention and that generates observable processes and outcomes. It is assumed that causality can be demonstrated if an activated mechanism is sufficient to generate an outcome of interest – if it actually operates in a specific context, the mechanism will produce the outcome of interest. This approach differs from the experimentalist’s view, which typically assumes that a causal variable influences the probability of having a particular outcome. Perhaps more importantly, mechanism-oriented approaches adopt a case-based approach to research, acknowledging that the complex causation processes underlying change in social systems requires a holistic perspective. Indeed, the entities that matter for demonstrating causality are not variables, but systems of agents and their relations. It would therefore be “*a fundamental ontological error to reify variables in abstraction from systems*” [14] (p. 114).

Critical realism [15] and scientific realism are among the main mechanism-oriented approaches. The latter inspired Pawson and Tilley to develop the realist evaluation approach [16]. Pawson and Tilley argued that in order to be useful for policymakers, managers and service providers, evaluations need to identify ‘what works in which conditions, for whom and why?’, rather than merely ‘does it work? [17] Realist evaluation belongs to the theory-driven inquiry school: realist evaluations start with a theory and end with a theory. Theory is to be understood as middle-range theories or theories of the middle range as defined by Merton [18] p. 39. These are “*theories that lie between the minor but necessary working hypotheses (…) and the all-inclusive systematic efforts to develop a unified theory that will explain all the observed uniformities of social behavior, social organization and social change*”.

**Fig. 1:**
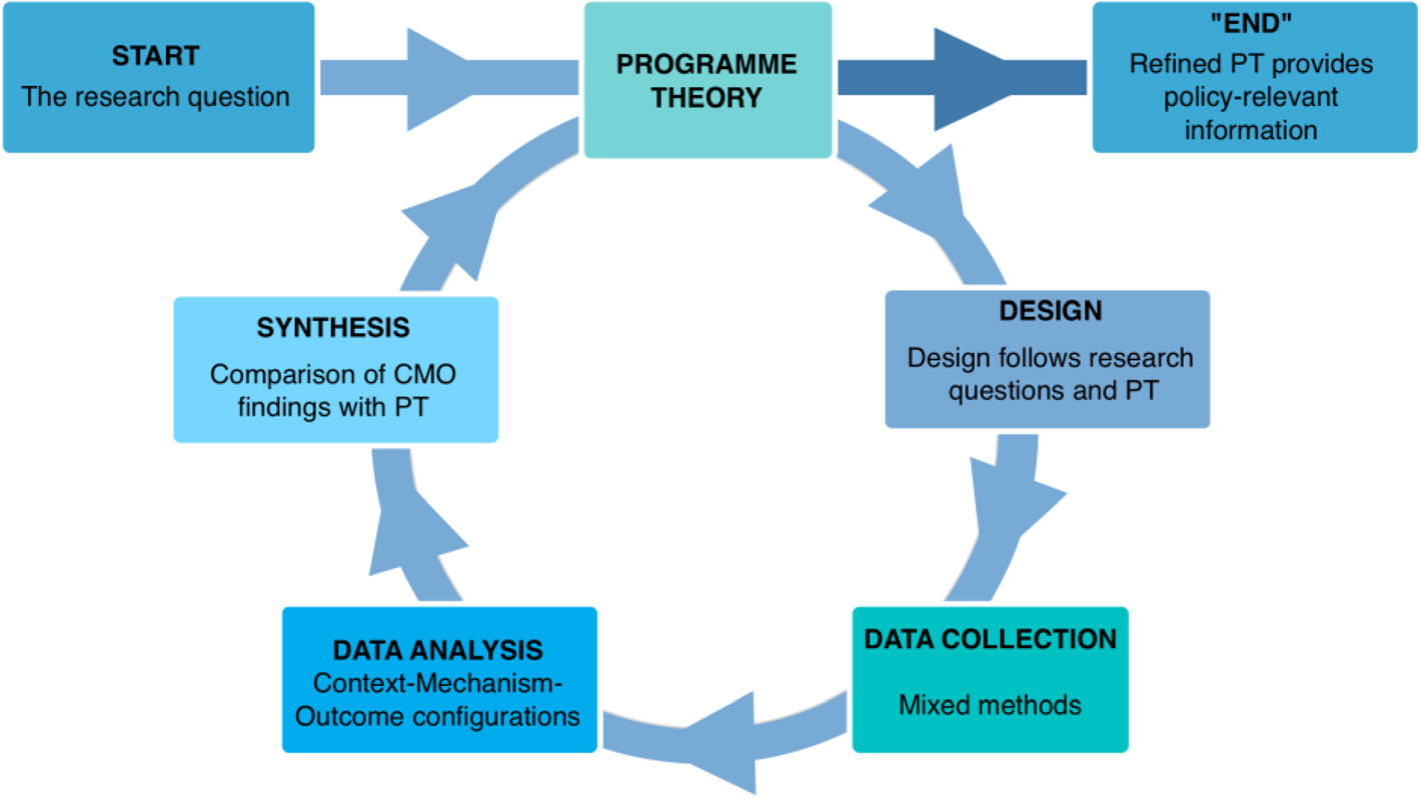
The realist evaluation cycle adapted from Marchal et al. [39]

The program theory (PT) or middle range theory - we will use the term PT in this paper - describes through which mechanisms the intervention is expected to lead to the outcomes and in which conditions it would do so. It is thus a detailed hypothesis that can be tested and refined at the end of the study. Realist evaluators aim at identifying the underlying generative mechanism that explains ‘how’ the outcome was caused and the context in which the mechanism is triggered. The issue of mechanisms is a subject of continued debate in realist circles. Pawson and Tilley define mechanisms as generally involving the underlying social or psychological drivers of the actors’ reasoning in response to the resources or opportunities provided by the intervention, and it is this what causes the outcomes [17]. Because RE embraces causal complexity [16], it is a promising approach to investigate complex interventions. Realist evaluations can be designed around the cycle presented by Fig. 1.

In a first step, the program theory is elicited by identifying the underlying assumptions about the intervention, its expected impact, the mechanisms that explain change and the required context conditions. This process is based on reviews of project documents, relevant literature and evaluations of similar interventions, as well as interviews with designers, implementers and ‘beneficiaries’.

In a second step, the choice of design and data collection methods is made. RE is method neutral. Realist studies are not driven by methodological preferences, but by the initial program theory or hypothesis: the design and data collection methods need to enable ‘testing’ the hypothesis. In practice, realists working in the field of health systems often adopt the case study design, with purposive sampling of cases and respondents and the collection of both quantitative and qualitative data.

Realist data analysis is driven by realist principles: realist researchers seeks to explain the observed outcomes of an intervention by referring to the actors who act and change (or not) a situation under specific conditions and under the influence of external events. The actors and the interventions are considered to be embedded in a social reality, which influences how the intervention is implemented and how actors respond to it (or not), and thus, which mechanisms are triggered. The Context-Mechanism-Outcome configuration is used as a tool to analyze the data. This means that the analysis aims at demonstrating how the observed outcomes can be explained by the interaction between intervention, (the social practices of the) actors, context and the mechanisms which underlie the social practices. In the last step, the CMO found to be the best explanation of the observed outcome is compared with the initial program theory, which is refined or adapted if necessary. (We refer to [16, 17, 19] and the Better Evaluation website http://www.betterevaluation.org/approach/realist_evaluation for a more detailed presentation of realist evaluation).

Using the realist evaluation approach to examine the relationship between participation and action of women groups and antenatal outcomes would enable evaluators to examine in detail the underlying mechanisms which influence actual practices and outcomes, as well as the context conditions required to make it work.

The study would start with extensive literature reviews on themes such as community participation, empowerment, social capital, social networks, group dynamics, power, etc. There is an abundance of literature on theories of participation. For participation, many of the most important theories are discussed in a recent World Bank review entitled “Localizing Development; does participation work?” [20]. Another starting point would be the review of WHO of the research on participatory women’s groups [21]. This report concluded that there was moderate evidence from the intervention on the reduction of newborn mortality in rural areas with limited health services, but also identified areas for investigation that included the presence of local social networks to support the intervention, the need for facilitators with good training and support; structures, systems and processes to make the interventions sustainable and the presence of socio-cultural barriers.

The researchers would also analyze evaluations of similar projects and review the program documents of the interventions under study. They could do exploratory interviews with the program designers, funders and implementers. The assumptions of the key stakeholders would then be compared with the published evidence and potentially important context conditions and mechanisms would be identified. This would allow them to formulate the initial program theory (PT).

On the basis of this initial PT, the study design would be selected. It would make sense to adopt a multiple case study design. In realist research, the cases are selected purposively: they need to enable testing of the program theory. That means that cases are selected on the basis of variation in context, outcome or intervention. In this case, one could start with four sites in each country.

The initial program theory would guide the choice of data collection tools, which would most likely include quantitative and qualitative data collection techniques to capture the key constructs of the PT. In realist evaluations, quantitative data are often used to document the outcomes and elements of the context, and qualitative data to identify the mechanisms at play. Attention would be paid to describe the actually implemented program in each study site and identify the actors involved in the implementation, including detailing the subgroups of participants. Once such patterns of outcomes are identified, the actors involved could be described and the mechanisms generating the outcomes identified and analyzed. The contexts in which particular mechanisms did or did not ‘fire’ could then be determined. Relevant context elements related to the sub-groups for whom outcomes were generated and/or to other stakeholders may include local and distal organizational, socio-economic, cultural and political conditions. The end result would be Intervention-Context-Actor-Mechanism-Outcome configurations in which qualitative and quantitative data are interwoven, and which would be formulated like “In context C1, where intervention I1 was implemented, mechanism M1 fired for actors A1, generating outcomes O1. In context C2, intervention I2 triggered mechanism M2, generating outcome O2 for actors A2. …” The comparison of findings from the different sites would then lead to refining the initial program theory. Ideally, this refined PT would be tested in another round of case studies. Such series of contrasting cases leads to specifying the program theory in a gradual process, extending its explaining power.

## Discussion

It should be noted that realist evaluation is not a magic bullet. Marchal and colleagues identified key concerns based on a systematic review of the literature. They noted a lack of consensus on some key concepts (such as mechanism) and highlighted the challenge of the demands of bringing to the evaluation the necessary methodological and substantive expertise and the commitment of time to investigate the intervention [21]. Since that review, however, a number of protocols of realist evaluations [22–26] and empirical studies [27–32] that can guide realist evaluations have been published. Guidance and reporting standards for realist evaluation have been published, too [33, 34].

In the meanwhile, the debate on how quasi-experimental designs can be combined (or not) with RE has started [35–38]. At the core of the debate is the issue whether the philosophical basis underpinning the (quasi-)experimental designs can accommodate a mechanism-oriented approach such as realist evaluation. More practically, the question is whether a RCT can identify mechanisms. From our perspective, the RCT design does not allow the researcher to identify the dynamic interplay between intervention, actors, contexts, mechanisms and outcomes, and thus, the term ‘realist RCT’ is a misnomer. That does not mean that RCTs have no place in research and evaluation of complex interventions. It means that the RCT should be used for what it is meant: to assess the effectiveness of interventions.

## Conclusions

In conclusion, the realist approach has the potential of making a significant contribution to the understanding of complex interventions that aim at health improvements. While realist research has been carried out on health service delivery, it has yet to be applied as a framework to exploring the role of community participation to health improvements. Through its focus on human behavior and context, realist research could open the “black boxes” of “community” and “participation” in order to examine the role they play in ensuring cost-effective, sustainable interventions.

## Abbreviations

CMO: Context-Mechanism-Outcome
MRC: Medical Research Council (UK)
PLA: Participatory learning and action
PT: Program theory
RCT: Random Control Trials
RE: Realist Evaluation
WHO: World Health Organization
